# Children with COVID-19 Who Manifest Febrile Seizure

**DOI:** 10.1155/2021/9992073

**Published:** 2021-06-24

**Authors:** Lilia Dewiyanti, Neni Sumarni, Joseph Deni Lie, Zuhriah Hidajati, Harancang Pandih Kahayana, Adriana Lukmasari, Cipta Pramana

**Affiliations:** ^1^Department of Pediatrics, K.R.M.T. Wongsonegoro General Hospital Semarang, Medical Faculty Tarumanagara University, Jakarta, Indonesia; ^2^Department of Obstetrics and Gynecology, K.R.M.T. Wongsonegoro General Hospital Semarang, Medical Faculty Tarumanagara University, Jakarta, Indonesia

## Abstract

The COVID-19 pandemic is a challenge for all medical personnel in the world. Various studies have been conducted to gain more knowledge about SARS-CoV-2, but studies in the pediatric population are still very limited. We report a case of a boy aged two years and seven months who came to the hospital with an atypical generalized seizure for less than 5 minutes and immediately regained consciousness after the seizure. Other symptoms included fever, productive cough, rhinorrhea, and shortness of breath. The X-ray showed a well-defined homogeneous consolidation in the upper right lobe and a small spot in both lungs which consistently showed top right lobar pneumonia and bronchopneumonia. From the SARS-CoV-2 nucleic acid test, positive results were obtained on the third day of hospitalization. The patient received antiseizure therapy, antibiotics, and other supportive therapies by Indonesian Pediatrician Association (IDAI) guidelines. During treatment, the patient responded well to the treatment given, with no other seizure episodes. A negative result on the SARS-CoV-2 nucleic acid test was obtained after twelve days of hospitalization as well as improvements of the lungs as seen from the X-ray.

## 1. Introduction

Severe acute respiratory syndrome (SARS) has long been studied in relation to coronavirus which originated from bats. SARS-CoV-2 belongs to the broad family of viruses known as coronaviruses, a group of very diverse viruses with enveloped, positive-sense single-stranded RNA [1]. The disease caused by SARS-CoV-2 was given the name COVID-19 by the WHO on February 11, 2020. SARS-CoV-2 originated in Wuhan, China, in December 2019 and has become a pandemic that has spread throughout the country. The clinical manifestations of COVID-19 are very diverse, including fever, dry cough, fatigue, headache, hemoptysis, diarrhea, and dyspnea. Patients with severe symptoms may develop pneumonia, acute respiratory distress syndrome, organ failure, and heart attack [2, 3]. Children with severe COVID-19 infection, especially those with multisystemic inflammation, have a higher risk of neurologic complications, which usually become the main reason for hospital visitation and admission [4]. Research by Bhatta et al. [5] in the USA reported a case about an eleven-year-old Hispanic boy who presented to the hospital with new-onset seizures. After the SARS-CoV-2 PCR test, a positive result was obtained and believed to be the cause of the seizure [5]. Based on current evidence, there is no age limit for COVID-19 susceptibility. Children, especially infants, are believed to be more susceptible to infectious diseases than adults because of the immaturity of their immune systems [6].

Epidemiological data of cumulative COVID-19 cases in Indonesia in December 2020 reached 664,930 cases. As many as 2.72% of positive cases of COVID-19 in Indonesia were children aged 0–5 years [7]. Cases of SARS-CoV-2 infection in preterm neonates in Semarang, Indonesia, were first reported on April 3, 2020, with severe respiratory problems. Their condition improved after receiving treatment in the hospital for 31 days [8]. Studies about COVID-19 in children are still very limited, but the possibility of various atypical symptoms and their variations cannot be avoided. More studies are needed to evaluate the impact of SARS-CoV-2 on children.

## 2. Case Report

**Figure 1:**
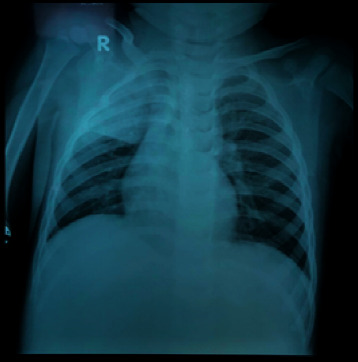
X-ray showing slight spotting in both lungs and homogeneous consolidation in the right upper lung with firm borders consistent with superior right lobar pneumonia and bronchopneumonia features.

A boy aged 2 years and 7 months came to the pediatric department with a generalized seizure for less than 5 minutes and immediately regained consciousness after the seizure. The child had a fever four days ago, continuous throughout the day, and was given a fever reducer. Other complaints were nonproductive cough, rhinorrhea, and shortness of breath two days before being admitted to the hospital. The patient also had diarrhea three times, but apart from the existing complaints, there were no other complaints such as sore throat, nausea, vomiting, stomach pain, and no clear history of COVID-19 exposure. The patient had a history of seizure at the age of six months, one year, and one and a half years and no history of tuberculosis infection and choking. On physical examination, the child looked weak and compos mentis; vital signs were as follows: pulse 120 x/min, respiratory rate 28 x/min, temperature 38°C, 98% oxygen saturation, cold extremities, and vesicular breath sound on thoracic auscultation found weakened at the upper right lung, no rhonchi, and no wheezing. From laboratory examination, the following data were acquired: haemoglobin level 11.8 g/dL, hematocrit 32.8%, leukocytes 27 × 10^9^/L, thrombocyte 341 × 10^9^/L, segment neutrophils 73.8%, lymphocytes 18.2%, NLR 4.1, and absolute lymphocyte count 4914/mm^3^. From blood gas analysis, the following data were acquired: pH 7.39, PCO_2_ 24 mmHg, PO_2_ 77 mmHg, HCO_3_ 14.5 mmol/L, and base excess −9.2 mmol/L. An X-ray radiograph showed consolidation of the right upper lung (Figure 1) which represented superior right lobar pneumonia and bronchopneumonia. The result of the first SARS-CoV-2 nucleic acid test on the day the child was admitted was negative, but one day later, on the second test, the result was positive. The patient received antiseizure therapy, antibiotics, nebulization, and symptomatic supportive therapy. A nasal cannula was supported with two liters of oxygen per minute and intravenous fluid. The patient responded well to the therapy given and recovered in 12 days.

## 3. Discussion

Clinical manifestations of COVID-19 in pediatrics can vary, ranging from asymptomatic, symptomatic, up to atypical symptoms. Guidelines made by the Indonesian Pediatrician Association (IDAI) classified COVID-19 clinically into six types: asymptomatic, mild symptoms, moderate symptoms, severe symptoms, critical symptoms, and multi-inflammatory syndrome [9]. The clinical classification of each degree varies. Clinically asymptomatic is defined as a case with a positive SARS-CoV-2 test without any clinical signs and symptoms. The mild degree is defined as a case with airway symptoms, fever, cough, rhinorrhea, sore throat, nausea, vomiting, abdominal pain, and diarrhea. A moderate degree is defined as a case with clinical signs and symptoms of pneumonia accompanied by rhonchi or wheezing. A severe degree is defined as a case with clinical features of severe pneumonia with nasal flaring, cyanosis, subcostal retraction, and oxygen desaturation <92% [9].

The patient was a boy aged 2 years and 7 months with atypical clinical manifestations, simple febrile seizure. Positive SARS-CoV-2 nucleic acid test and a well-defined homogeneous consolidation in the upper right lung (features of superior right lobar pneumonia and bronchopneumonia) from X-ray imaging were obtained on December 31, 2020. This patient is classified as a COVID-19 patient with moderate symptoms. The patient underwent isolation and received antiseizure therapy (intravenous diazepam injection: 0.5 mg/Kg BW), empiric antibiotic therapy (IV ceftriaxone injection: 80 mg/Kg BW/24 hours and IV azithromycin injection: 10 mg/Kg BW), and nebulized ipratropium bromide/salbutamol sulphate + fluticasone propionate. The patient responded well to the therapy given, and SARS-CoV-2 nucleic acid test was negative after 12 days of treatment. Pediatric patients can recover under standard therapy according to IDAI guidelines without being given antivirals.

**Figure 2:**
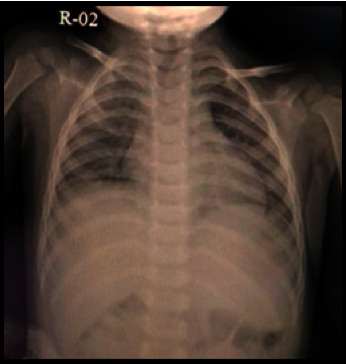
X-ray showing lung improvement.

X-rays of pediatric patients who are tested positive for COVID-19 can be normal. The most common findings are peribronchial cuffing and perihilar opacity. The distribution of abnormalities can be unilateral or bilateral with a predominance of the peripheral and lower lung area. The appearance of lobar pneumonia is an atypical presentation of COVID-19 [10]. In the case we found, namely, a child aged two years and seven months with atypical features, upper right lobar pneumonia recovered as seen through X-ray imaging on January 11, 2021 (Figure 2).

Patients infected with coronavirus and showing respiratory symptoms may be associated with neurological complaints. After infecting the nasal airway, virus particles can enter the central nervous system via the olfactory tract, causing inflammation and demyelination. Once the infection has settled, the coronavirus can reach the brain and cerebrospinal fluid in less than seven days. Neurological manifestations in patients with coronavirus include febrile seizures, convulsions, altered mental status, encephalomyelitis, and encephalitis [3]. A study conducted by Li et al. [11] in Hunan, China, showed that 22 out of 183 pediatric patients with acute encephalitis were tested positive for anti-CoV IgM antibodies [11].

Anam et al. [12] compared the clinical profile of children with COVID-19 at RSUP Kariadi Semarang, Central Java, Indonesia. Demographic characteristics of children infected with COVID-19 are as follows: dominated by male patients (53.7%), with the greatest age range of 1–5 years (43.9%), and without a definite history of COVID-19 exposure (85.4%). The clinical manifestations of children with COVID-19 are fever (90.2%), cough (92.7%), rhonchi (61%), symptoms other than respiratory symptoms (51.8%), costal retraction (34.1%), shortness of breath (26.8%), nausea/vomiting (26.8%), diarrhea (24.4%), rhinorrhea (22%), fatigue (19.5%), and wheezing (14.6%). The laboratory data are as follows: anemia (34.1%), leukopenia (12.1%), leucocytosis (29.2%), thrombocytopenia (24.4%), thrombocytosis (22%), lymphopenia (22%), and lymphocytosis (4.8%). X-ray imaging findings are as follows: without infiltrates (2.4%), unilateral infiltrates (36.5%), bilateral infiltrates (51.2%), and consolidation (34.1%) [12]. These are consistent with the case we found, a child aged 2 years and 7 months, with complaints of fever, productive cough, rhinorrhea, shortness of breath, and diarrhea and without a clear history of COVID-19 exposure in Semarang. However, the patient in our report sought medical attention due to simple febrile seizures. Another limitation in our report is that electroencephalography and CT scan were not performed.

## 4. Conclusion

The reported case of a child aged 2 years and 7 months with clinical manifestations of febrile seizures suspected to be caused by COVID-19 was a unique case and an atypical presentation that clinicians should pay attention to. Management of simple febrile seizures in children and finding the cause must be initiated immediately. Pediatric patients infected with COVID-19 do not always come to the hospital with respiratory or gastrointestinal symptoms. We suggest considering COVID-19 to be screened in pediatric patients with febrile seizure.

## Data Availability

The data used to support the findings of this study are available from the corresponding author upon request.
